# Supplementary material for: Heat adaptation of phage T7 under an extended genetic code
Source: Virus Evol. 2021 Dec 1;7(2):veab100. doi: 10.1093/ve/veab100 (PMC8923235; doi:10.1093/ve/veab100)
Supplement: veab100_Supp [file veab100_supp.zip › Sequencing_Analysis.zip!/Sequencing_Analysis/A2/evidence/JC_SIDE_1_46.html]

BRESEQ :: Evidence


| New junction evidence | | | | | | | | | | | |
| --- | --- | --- | --- | --- | --- | --- | --- | --- | --- | --- | --- |
|  | | seq id | position | reads (cov) | reads (cov) | score | skew | freq | annotation | gene | product |
| \* | ? | NC\_001604.1 | = 7613 | 539 (1.180) | 7 (0.020) | 3/586 | 23.5 | 1.3% | intergenic (–/–) | *–/–* | –/– |
|  | ? | NC\_001604.1 | 7912 = | 522 (1.170) |  |  |  |  | intergenic (–/–) | *–/–* | –/– |
| Rejected: Coverage evenness skew score above cutoff. | | | | | | | | | | | |
| Rejected: Frequency below/above cutoff threshold. | | | | | | | | | | | |

Only 100 of 542 total aligned reads displayed.  

`CCACGAATATCTCTCGCGCCTTAATGGATGAGTTCACTGAGACAGTAAAAGAGGCCACCCTAAGTCAATGGGGATTCTTTAGCCCATACGGTATTGGCGACAACGATGCTTGTACTATTAACCCTTACGATGGCTGGGCGTGTCAAATTAGCTACATGGAGGAAACACCTGATGGCTCTTTGCGGCACCCATCGTTCGTAATGTTCCGTGGCACCGA‑GGACAACCCTCAAGAGAAAATGTAATCACACTGGCTCACCTTCGGGTGGGCCTTTCTGCGTTTATAAGGAGACACTTTATGTTTAAGAAGGTTGGTAAATTCCTTGCGGCTTTGGCAGCTATCCTGACGCTTGCGTATATTCTTGCGGTATACCCTCAAGTAGCACTAGTAGTAGTTGGCGCTTGTTACTTAGCGGCAGTGTGTGCTTGCGTGTGGAGTATAGTTAACTGGTAATACGACTCACTAAAGGAGGTACACACCATGATGTACTTAATGCCATTACTCATCGTCATTGTAGGATGCCTTGCGCTCCACTGTAGCGATGATGATATGCCAGATGGTCACGCTTAATACGACTCACTAAAGGAGACACTATATGTTTC  >  NC_001604.1/7313‑7912`  

                                                                                                                                                                                                                                                                                                             `|`                                                                                                                                                                                                                                                                                                             
                  `cgagagtctcgtgggctcggagatgtgTATAAGAGACAGCCCTAAGTCAATGGGGATTCTTTAGCCCATACGGTATTGGCGACAACGATGCTTGTACTATTAACCCTTACGATGGCTGGGCGTGTCAAATTAGCTACATGGAGGAAACACCTGATGGCTCTTTGCGGCACCCATCGTTCGTAATGTTCCGTGGCACCGA‑GGACAACCCTCAAGAGAAAATGTAATCACACTGGCTCACCTTCGGGTGGGCCTTTCTGCGTTTATAAGGAGACACTTTATGTTTAAGAAGGTTGGTAAATTc                                                                                                                                                                                                                                                                                           <  1:532040/275‑1 (MQ=255)`  
                     `tAATGGATGAGTTCACTGAGACAGTAAAAGAGGCCACCCTAAGTCAATGGGGATTCTTTAGCCCATACGGTATTGGCGACAACGATGCTTGTACTATTAACCCTTACGATGGCTGGGCGTGTCAAATTAGCTACATGGAGGAAACACCTGATGGCTCTTTGCGGCACCCATCGTTCGTAATGTTCCGTGGCACCGA‑GGACAACCCTCAAGAGAAAATGTAATCACACTGGCTCACCTTCGGGTGGGCCTTTCTGCGTTTATAAGGAGACACTTTATGTTTAAGAAGGTTGGTAAATTCCtg                                                                                                                                                                                                                                                                                        >  1:48101/1‑300 (MQ=255)`  
                     `tAATGGATGAGTTCACTGAGACAGTAAAAGAGGCCACCCTAAGTCAATGGGGATTCTTTAGCCCATACGGTATTGGCAACAACGATGCTTGTACTATTAACCCTTACGATGGCTGGGCGTGTCAAATTAGCTACATGGAGGAAACACCTGATGGCTCTTTGCGGCACCCATCGTTCGTAATGTTCCGTGGCACCGA‑GGACAAACCTCAAGAGAAAATGTAATCACACTGGATCACCTTCGGGTGGGCCTTTCTGCGTTTATAAGGAGACACTTTATGTTTAAGAAGGTTggctgtctctta                                                                                                                                                                                                                                                                                        >  1:542597/1‑291 (MQ=255)`  
                       `aTGGATGAGTTCACTGCGACAGTAAAAGAGGCCACCCTAAGTCAATGTGGATTCTTTAGCCCATACGGTATTGGCGACAACGATGCTTGTACTATTAACCCTTACGATGGCTGGGCGTGTCAAATTAGCTACATGGAGGAAACACCTGATGGCTCTTTGCGGCACCCATCGTTCGTAATGTTCCGTGGCACCGA‑GGACAACCCTCAAGAGAAAATGTAATCACACTGGCTCACCTTCGGGTGGGCCTTTCTGCGTTTATACGGAGACACTTTATGtttcgacttcattacaacaaaagctt                                                                                                                                                                                                                                                                                      >  1:257474/1‑278 (MQ=255)`  
                               `gTTCACTGAGACAGTAAAAGAGGCCACCCTAAGTCAATGGGGATTCTTTAGCCCATACGGTATTGGCGACAACGATGCTTGTACTATTAACCCTTACGATGGCTGGGCGTGTCAAATTAGCTACATGGAGGAAACACCTGATGGCTCTTTGCGGCACCCATCGTTCGTAATGTTCCGTGGCACCGA‑GGACAACCCTCAATAGAAAATGTAATCACACTGGCTCACCTTCGGGTGGGCCTTTCTGCGTTTATAAGGAGACACTTTATGTTTAAGAAGGtctgtctcttatacacctctccga                                                                                                                                                                                                                                                                              >  1:744725/1‑278 (MQ=255)`  
                               `gTTCACTGAGACAGTAAAAGAGGCCACCCTAAGTCAATGGGGATTCTTTAGCCCATACGGTATTGGCGACAACGATGCTTGTACTATTAACCATTACGATGGCTGGGCGTGTCAAATTAGCTACATGGAGGAAACACCTGATGGCTCTTTGCGGCACCCATCGTTCGTAATGTTCCGTGGCACCGA‑GGACAACCATCAAGAGAAAATGTAATCACACTGGCTCACCTTCGGGTGGGCCTTTCTGCGTTTATAAGGAGACACTTTATGTTTAAGAAGGTTGGTAAATTACTTGCGGCTTTgg                                                                                                                                                                                                                                                                              >  1:363064/1‑301 (MQ=255)`  
                                `ttCACTGAGACAGTAAAAGAGGCCACCCTTAGTCAATGGGGATTCTTTAGCCCATTCGGTATTGGCGACAACGATGCTTGTACTATTAACCCTTACGATTGCTGGGCGTGTCAAATTAGCTACATGGAGGAAACACCTGATGGCTCTTTGCGGCACCCATCGTTCGTAATGTTCCGTGGCACCGA‑GGACAACCCTCAAGAGAAAATGTAATCACACTGGCTCACCTTCGGGTGGGCCTTTCTGCGTTTATAAGGAGACACTTTATGTTTAAGAAGGTTGGTAAATTCCTTGCGGCTTTGGc                                                                                                                                                                                                                                                                             <  1:74186/301‑1 (MQ=255)`  
                                  `cccTGAGACAGTAAAAGAGGCCAACCTAAGTCAATGGGGATTCTTTAGCCCATACGGTATTGGCGACAACGATGCTTGTACTATTAACCCTTACGATGGCTGGGCGTGTCAAATTAGCTACATGGAGGAAACACCTGATGGCTCTTTGCGGCACCCATCGTTCGTAATGTTCCGTGGCACCGA‑GGACAACCCTCAAGAGAAAGTGTAATCACACTGGCTCACCTTCGGGTGGGCCTTTCTGCGTTTATAAGGAGACACTTTATGTTTAAGAAGGTTGGTAAATTCCTTGCGGCTTTGGCAg                                                                                                                                                                                                                                                                           <  1:508398/299‑1 (MQ=255)`  
                                  `cACTGAGACAGTAAAAGAGGCCACCATAAGTCAATGGGGATTCTTTAGCCCATACGGTATTGGCGACAACGATGCTTGTACTATTAACCCTTACGATGGCTAGGCGTGTCAAATTAGCTACATGGAGGAAACACCTGATGGCTCTTTGCGGCACCCATCGTTCGTAATGTTCCGTGGCACCGA‑GGACAACCCTCAAGAGAAAATGTAATCACACTGGCTCACCTTCGGGTGGGCCTTTCTGCGTTTATAAGGAGACACTTTATGTTTAAGAAGGTTGGTAAATTCCTTGCGGCTTTGGCAg                                                                                                                                                                                                                                                                           >  1:87982/1‑301 (MQ=255)`  
                                            `gTACAAGAGGCCACCCTAAGTCAATGGGGATTCTTTAGCCCATACGGTATTGGCGACAACGATGCTTGTACTATTAACCCTTACGATGGCTGGGCGTGTCAAATTAGCTACATGTAGGAAACACCTGATGGCTCTTTGCGGCACCCATCGTTCGTAATGTTCCGTGGCACCGA‑GGACAACCCTCAAGAGAAAATGTAATCACACTGGCTCACCTTCGGGTGGGCCTTTCTGCGTTTATAAGGAGACACTTTATGTTTAAGAAGGTTGGTAAATTCCTTGCGGCTTTGGCAGCTATCCTGAc                                                                                                                                                                                                                                                                 >  1:296655/1‑301 (MQ=255)`  
                                             `tAAAAGAGGCCACCCTAAGTCAATGGGGATTCTTTAGCCCATACGGTATTGGCGACAACGATGCTTGTACTATTAACCCTTACGATGGCTGGGCGTGTCAAATTAGCTACATGGAGGAAACACCTGATGGCTCTTTGCGGCACCCATCGTTCGTAATGTTCCGTGGCACCGA‑GGACAACCCTCAAGAGAAAATGTAATCACACTGGCTCACCTTCGGGTGGGCCTTTCTGCGTTTATAAGGAGACACTTTATGTTTAAGAAGGTTGGTAAATTCCTTGCGGCTTTGGCAGCTATCCTGACg                                                                                                                                                                                                                                                                <  1:390476/301‑1 (MQ=255)`  
                                              `aaaaGAGGCCACCCTAAGTCAATGGGGATTCTTTAGCCCATACGGTATTGGCGACAACGATGCTTGTACTATTAACCCTTACGATGGCTGGGCGTGTCAAATTAGCTACATGGAGGAAACACCTGATGGCTCTTTGCGGCACCCATCGTTCGTAATGTTCCGTGGCACCGA‑GGACAACCCTCAATAGAAAATGTAATCACACTGGCTCACCTTCGGGTGGGCCTTTCTGCGTTTATAAGGAGACACTTTATGTTTAAGAAGGTTGGTAAATTCCtgtctcttatacacatctccgagccca                                                                                                                                                                                                                                                               >  1:784371/1‑275 (MQ=255)`  
                                                     `gggagatgtgtataagagacagtCTTTAG‑CCATACGGTATTGGCGACAACGATGCTTGTACTATTAACCCTTACGATGGCTGGGCGTGTCAAATTAGCTACATGGAGGAAACATCTGATGGCTCTTTGCGGCACCCATCGTTCGTAATGTTCCGTGGCACCGA‑GGACAACCCTCAATAGAAAATGTAATCACACTGGCTCACCTTCGGGTGGGCCTTTCTGCGTTTATAAGGAGACACTTTATGTTTAAGAAGGTTGGTAAATTCCTTGCGGCTTTGGCAGCTATCCTGACGCTTGCGtat                                                                                                                                                                                                                                                       <  1:553596/279‑1 (MQ=255)`  
                                                         `cccTAAGTCAATGGGGATTCTTTAGCCCATACGGTATTGGCGACAACGATGCTTGTACTATTAACCCTTACGATGGCTGGGCGTGTCAAATTAGCTACATGGAGGAAACACCTGATGGCTCTTTGCGGCACCCATCGTTCGTAATGTTCCGTGGCACCGA‑GGACAACCCTCAAGAGAAAATGTAATCACACTGGCTCACCTTCGGGTGGGCTTTTCTGCGTTTATAAGGAGACACTTTATGTTTAAGAAGGTTGGTAAATTCCTTGCGTCTTTGGCAGCTATCCTGACGCTTGCGTATAtt                                                                                                                                                                                                                                                    >  1:523422/1‑301 (MQ=255)`  
                                                         `cccTAAGTCAATGGGGATTCTTTAGCCCATACGGTATTGGCGACAACGATGCTTGTACTATTAACCCTTACGATGGCTGGGAGTGTCAAATTAGCTACATGGAGGAAACACCTGATGGCTCTTTGCGGCACCCATCGTTCGTAATGTTCCGTGGCACCGA‑GGACAACCCTCAAGAGAAAATGTAATCACAATGGCTCACCTTCGGGTGGGCCTTTCTGCGTTTACAAGGAGACACTTTATGTTTAAGAAGGTTGGTAAATTCCTTGCGGCTTTGGCAGCTATCCTGACGCTTGCGTATAtt                                                                                                                                                                                                                                                    >  1:367644/1‑301 (MQ=255)`  
                                                                `tCAATGGGGATTCTTTAGCCCATACGGTATTGGCGACAACGATGCTTGTACTATTAACCCTTACGATGGCTGGGCGTGTCAAATTAGCTACATGGAGGAAACACCTGATGGCTCTTTGCGGCACCCATCGTTCGTAATGTTCCGTGGCACCGA‑GGACAACCCTCAAGAGAAAATGTAATCACACTGGCTCACCTTCGGGTGGGCCTTTCTGCGTTTATAAGGAGACACTTTATGTTTAAGAAGGTTGGTAAATTCCTTGCGGCTTTGGCAGCTATCCTTACGCtgtctcttatacacatct                                                                                                                                                                                                                                             >  1:132136/1‑284 (MQ=255)`  
                                                                     `agggATTCTTTAGCCCATACGGTATTGGCGACAACGATGCTTGTACTATTAACCCTTACGATTGCTGGGCGTGCCAAATTAGCTACATGGAGGAAACACCTGATGGCTCTTTGTGGCACCCATCGTTCGTAATGTTCCGTGGCACCGA‑GGACAACCCTCAAGAGAAAATGTAATCACACTGGCTCACCTTCGGGTGGGCCTTTCTGCGTTTATAAGGAGACACTTTATGTTTAAGAAGGTTGGTAAATTCCTTGCGGCTTTGGCAGCTATCCTGACGCTTGCGTATATTCTTGCAGTATAc                                                                                                                                                                                                                                        <  1:116251/300‑1 (MQ=255)`  
                                                                        `tataagagacagcATATGGTATTGGCGACAACGATGCTTGTACTATTAACCCTTACGATGGCTGGGCGTGTCAAATTAGTTACATGGAGGAAACACCTGATGGCTCTTTGCGGCACCCATCGTTCGTAATGTTCCGTGGCACCGA‑GGACAACCCTCAAGAGAAAATGTAATCACACTGGCTCACCTTCGGGTGGGCCTTTCTGCGTTTATAAGGAGACACTTTATGTTTAAGAAGGTTGGTAAATTCCTTGCGGCTTTGGCAGCTATCCTGACGCTTGCGTATATTCTTGCGGTATACCCt                                                                                                                                                                                                                                     <  1:503694/289‑1 (MQ=255)`  
                                                                        `gATTCTTTAGCCCATACGGTATTGGCGACAACGATGCTTGTACTATTAACCCTTACGATGGCTGGGCGTGTCAAATTAGCTACATGGAGGAAACACCTGATGGCTCTTTGCGGCACCCATCGTTCGTAATGTTCCGTGGCACCTA‑GGACAACCCTCAAGAGAAAATGTAATCACACTGGCTCACCTTCGGGTGGGCCTTTCTGCGTTTATAAGGAGACACTTTATGTTTAAGAAGGTTGGTAAATTCCTTGCGGCTTTGGCAGCTATCCTGACGCTTGCGTATATTCTTGCGGTATACCCt                                                                                                                                                                                                                                     <  1:120610/301‑1 (MQ=255)`  
                                                                        `acagcattAGCCCATACGGTATTGGCGACAACGATGCTTGTACTATTAACCCTTACGATGGCTGGGCGTGTCAAATTAGCTACATGGAGGAAACACCTGATGGCTATTTGCGGCACCCATCGTTCGTAATGTTCCGTGGCACCGA‑GGACAACCCTCAAGAGAAAATGTAATCACACTGGCTCACCTTCGGGTGGGCCTTTCTGCGTTTATAAGGAGACACTTTATGTTTAAGAAGGTTGGTAAATTCCTTGCGGCTTTGGCAGCTATCCTGACGCTTGCGTATATGCTTGCGGTATACCCt                                                                                                                                                                                                                                     <  1:812472/295‑1 (MQ=255)`  
                                                                           `tCTTTAGCCCATACTGTATTGGCGACAACGATGCTTGTACTATTAACCCTTACCATGGCTGGGCGTGTCATATTAGCTACATGTAGGAAACACCTGATGGCTCTTTGCGGCACCCATCGTTCGTAATGTTCCGTGGCACCGA‑GGACAACCCTCAAGAGCAAATGTAATCACACTGGCTCACCTTCGGGTGGGCCTTTCTGCGTTTATAAGGAGACACTTTATGTTTAAGAAGGTTGGTAAATTCCTTGCGGCTTTGCCAGCTATCCTGACGCTTGCTTATATTCTTTCGGTATACCTTCaa                                                                                                                                                                                                                                  >  1:381862/1‑301 (MQ=255)`  
                                                                            `cTTTAGCCCATACGGTATTGGCGACAACGATGCTTGTACTATTAACCCTTACGATGGCTGGGCGTGTCAAATTAGCTACATGGAGGAAACACCTGATGGCTCTTTGCGGCACCCATCGTTCGTAATGTTCCGTGGCACCGA‑GGACAACCCTCAAGAGAAAATGTAATCACACTGGCTCACCTTCGGGTGGGCCTTTCTGCGTTTATAAGGAGACACTTTATGTTTAAGAAGGTTGGTAAATTCCTTTCGGCTTTGGCAGCTATCCTGACGCTTGCGTATATTCTTGCGGTATACCCTCAAg                                                                                                                                                                                                                                 >  1:280261/1‑301 (MQ=255)`  
                                                                             `tttAGCCCATACGGTATTGGCGACAACGATGCTTGTACTATTAACCCTTACGATGGCTGGGCGTGTCAAATTAGCTACATGGAGGAAACACCTGATGGCTCTTTGCGGCACCCATCGTTCGTAATGTTCCGTGGCACCGA‑GGACAACCCTCAAGAGAAAATGTAATCACACTGGCTCACCTTCGGGTGGGCCTTTCTGCGTTTATAAGGAGACACTTTATGTTTAAGAAGGTTGGTAAATTCCTTGCGGCTTTGGCAGCTATCCTGACGCTTGCGTATATTCTTGCGGTATACCCTCAAGt                                                                                                                                                                                                                                <  1:400967/301‑1 (MQ=255)`  
                                                                             `tttAGCCCATACGGTATTGGCGACAACGATGCTTGTACTATTAACCCTTACGATGGCTGGGCGTGTCAAATTAGCTACATGGAGGAAACACCTGATGGCTCTTTGCGGCACCCATCGTTCGTAATGTTCCGTGGCACCGA‑GGACAACCCTCAAGAGAAAATGTAATCACACTGGCTCACCTTCGGGTGGGCCTTTCTGCGTTTATAAGGAGACACTTTATGTTTAAGAAGGTTGGTAAATTCCTTGCGGCTTTGGCAGCTATCCTGACGCTTGCGTATATTCTTGCGGTATACCATCAAGt                                                                                                                                                                                                                                <  1:624179/301‑1 (MQ=255)`  
                                                                                 `gCCCATACGGTATTGGCGACAACGATGCTTGTACTATTAACCCTTACGATGGCTGGGCGTGTCAAATTAGCTACATGGAGGAAACACCTGATGGCTCTTTGCGGCACCCATCGTTCGTAATGTTCCGTGGCACCGA‑GGACAACCCTCAAGAGAAAATGTAATCACACTGGCTCACCTTCGGGTGGGCCTTTCTGCGTTTATAAGGAGACACTTTATGTTTAAGAAGGTTGGTAAATTCCTTGCGGCTTTGGCAGCTATCCTGACGCTTGCGTATATTCTTGCGGTATACCATCAAGTAGCa                                                                                                                                                                                                                            >  1:618290/1‑301 (MQ=255)`  
                                                                                 `gCCCATACGGTATTGGCGACAACGATGCTTGTACTATTAACCCTTACGATGGCTGGGCGTGTCAAATTAGCTACATGGAGGAAACACCTGATGGCTCTTTGCGGCACCCATCGTTCGTAATGTTCCGTGGCACCGA‑GAACAACCCTCAAGAGAAAATGTAATCACACTGGCTCACCTTAGGGTGGGCCTTTCTGCGTTTATAAGGAGACACTTTATGTTTAAGAAGGTTGGTAAATTCCTTGCGGCTTTGGCAGCTATCCTGACGCTTGCGTATATTCTTGCGGTATACGCTCactgtctc                                                                                                                                                                                                                            >  1:329728/1‑294 (MQ=255)`  
                                                                                  `gtgggctcggagatgtgtataagagacaggTACTATTAACCCTTACGATGGCTGGGCGTGTCAAATTAGCTACATGGAGGAAACACCTGATGGCTCTTTGCGGCACCCATCGTTCGTAATGTTCCGTGGCACCGA‑GGACAACCCTCAAGAGAAAATGTAATCACACTGGCTCACCTTCTGGTGGGCCTTTCTGCGTTTATAAGGAGACACTTTATGTTTAAGAAGGTTGGTAAATTCCTTGCGGCTTTGGCAGCTATCCTGACGCTTGCGTATATTCTTGCGGTATACCCTCAAGTAGCAc                                                                                                                                                                                                                           <  1:734667/272‑1 (MQ=255)`  
                                                                                  `ccaaTACGTTATTGGCGACAACGATGCTTGTACTATTAACCCTTACGATGGCTGGGCGTGTCAAATTAGCTACATGGAGGAAACACCTGATGGCTATTTGCGGCACCCATCGTTCGTAATGTTCCGTGGAACCGA‑GGACAACCCTCAAGAGAAAATGTAATCACACTGGCTCACCTTCGGGTGGGCCTTTCTGCGTTTATAAGGAGACACTTTATGTTTAAGAAGGTTGGTAAATTCCTTGCGGCTTTGGCAGCTATCCTGACGCTTGCGTATATTCTTGCGGTATACCCTCAAGTAGCAc                                                                                                                                                                                                                           <  1:711354/298‑1 (MQ=255)`  
                                                                                    `cATACGGTATTGGCGACAACGATGCTTGTACTATTAACCCTTACGATGGCTGGGCGTGTCAAATTAGCTACATGGAGGAAACACCTGATGGCTCTTTGCGGCACCCATCGTTCGTAATGTTCCGTGGCACCGA‑GGACAACCCTCAAGAGAAAATGTAATCACACTGGCTCACCTTCGGGTGGGCCTTTCTGCGTTTATAAGGAGACACTTTATGTTTAAGAAGGTTGGTAAATTCCTTGCGGCTTTGGCAGCTATCCTGACGCTTGCGTATATTCTTGCGGTATACCCTCAAGTAGCACta                                                                                                                                                                                                                         <  1:589363/301‑1 (MQ=255)`  
                                                                                     `aTACGGTATTGGCGACAACGATGCTTGTACTATTAACCCTTACGATGGCTGGGCGTGTCAAATTAGCTACATTGAGGAAACACCTGATGGCTCTTTGCGGCACCCATCGTTCGTAATGTTCCGTGGCACCGA‑GGACAACCCTCAAGAGAAAATGTAATCACACTGGCTCACCTTCGGGTGGGCCTTTCTGCGTTTATAAGGAGACACTTTATGTTTAAGAAGGTTGGTAAATTCCTTGCGGCTTTGGCAGCTATCCTGACGCTTGCGTATATTCTTGCGGtctgtctcttatacacatctc                                                                                                                                                                                                                        >  1:46431/1‑281 (MQ=255)`  
                                                                                     `aTACGGTATTGGCGACAACGATGCTTGTACTATTAACCCTTACGATGGCTGGGCGTGTCAAATTAGCTACATGGAGGAAACACCTGATGGCTCTTTGCGGCACCCATCGTTCGTAATGTTCCGTGGCACCGA‑GGACAACCCTCAAGATAAAATGTAATCACACTGGCTCACCTTCGGGTGGGCCTTTCTGCGTTTATAAGGAGACACTTTATGTTTAAGAAGGTTGGTAAATTCCTTGCGGCTTTGGCAGCTATCCTGACGCTTGCGTATATTCTTTCGGTATACCCTCAAGTAGCACtag                                                                                                                                                                                                                        <  1:764992/301‑1 (MQ=255)`  
                                                                                     `aTACGGTATTGGCGACAACGATGCTTGTACTATTAACCCTTACGATGGCTGGGCGTGTCAAATTAGCTACATGGAGGAAACACCTGATGGCTCTTTGCGGCACCCATCGTTCGTAATGTTCCGTGGCACCGA‑GGACAACCCTAAAGAGAAAATGTAATCACACTGGCTCACCTTCGGGTGGGCCTTTCTGCGTTTATAAGGAGACACTTTATGTTTAAGAAGGTTGGTAAATTCCTTGCGGCTTTGGCAGCTATACTGACGCTTGCGTATATTCTTGCGGTATACCATCAAGTAGCACtag                                                                                                                                                                                                                        >  1:699996/1‑301 (MQ=255)`  
                                                                                          `gTATTGGCGACAACGATGCTTGTACTATTAACCCTTACGATGGCTGTGCGTGTCAAATTAGCTACATGGAGGAAACACCTGATGGCTCTTTGCGGCACCCATCGTTCGTAATGTTCCGTGGCACCGA‑GGACAACCCTCAAGAGAAAATGTAATCACACTGGCTCACCTTCTGGTGGGCCTTTCTGCGTTTATAAGGAGACACTTTATGTTTAAGAAGGTTGGTAAATTCCTTGCGGCTTTGGCAGCTATCCTGACGCTTGCGTATATTCTTGCGGTATACCCTCAAGTAGCACtagtcgtt                                                                                                                                                                                                                   >  1:777427/1‑300 (MQ=255)`  
                                                                                          `gTATTGGCGACAACGATGCTTGTACTATTAACCCTTACGATGGCTGGGCGTGTCAAATTAGCTACATGGAGGAAACACCTGATGGCTCTTTGCGGCACCCATCGTTCGTAATGTTCCGTGGCACCGA‑GGACAACCCTCAAGATAAAATGTAATCACACTGGCTCACCTTCGGGTGGGCCTTTCTGCGTTTATAAGGAGACACTTTATGTTTAAGAAGGTTGGTAAATTCCTTGCGGCTTTGGCAGCTATCCTGACGCTTGCGTATATTCTTGTGGTATACCCTCAAGTAGCACtagtagta                                                                                                                                                                                                                   >  1:221477/1‑301 (MQ=255)`  
                                                                                          `gTATTGGCGACAACGATGCTTGTACTATTAACCCTTACGATGGCTGGGCGTGTCAAATTAGCTACATGGAGGAAACACCTGATGGCTCTTTGCGGCACCCATCGTTCGTAATGTTCCGTGGCACCGA‑GGACAACCCTCAAGAGAAAATGTAATCACACTGGCTCACCTTCGGGTGGGCCTTTCTGCGTTTATAAGTAGACACTTTATGTTTAAGAAGGTTGGTAAATTCCTTGCGGCTTTGGCAGCTATCCTGACGCTTGCGTATATTCTTGCGGTATACCCTCAAGTAGCACtagtagta                                                                                                                                                                                                                   >  1:14919/1‑301 (MQ=255)`  
                                                                                               `ggCGACAACGATGCTTGTACTATTAACCCTTACGATGGCTGGGCGTGTCAATTTAGCTACATTGAGGAAACACCTGATGGCTCTTTGCGGCACCCATCGTTCGTAATGTTCCGTGGCACCGA‑GGACAACCCTCAAGAGAAAATGTAATCACACTGGCCCACCTTCGGGTGGGTCTTTCTGCCTTTATAAGGAGACACTTTATGTTTAAGAAGGTTGGTAAATTCCTTGCGGCTTTGGCAGCTATCTTGACGCTTGCGTATATTCTTGCGGTATACCCTCAAGTAGCACTAGTAGTTGTTgg                                                                                                                                                                                                              >  1:30873/1‑301 (MQ=255)`  
                                                                                                          `tGCTTGTACTATTAACCCTTACGATGGCTGGGCGTGTCAAATTAGCTACATGGAGGAAACACCTGATGGCTCTTTGCGGCACCCATCGTTCGTAATGTTCCGTGGCACCGA‑GGACAACCCTCAAGAGAAAATGTAATCACACTGGCTCACCTTCGGGTGGGCCTTTCTGCGTTTATAAGGAGACACTTTATGTTTAAGAAGGTTGGTAAATTCCTTGCGGCTTTGGCAGCTATCCTGACGCTTGCGTATATTCTTGCGGTATACCCTCAAGTAGCACTAGTAGTAGTTGGCGCTTTTTTCt                                                                                                                                                                                                   >  1:318017/1‑301 (MQ=255)`  
                                                                                                          `tGCTTGTACTATTAACCCTTACGATGGCTGGGCGTGTCAAATTAGCTACATGGAGGAAACACCTGATGGCTCTTTGCGGCACCCATCGTTCGTAATGTTCCGTGGCACCGA‑GGACAACCCTCAAGAGAAAATGTAATCACACTGGCTCACCTTCGGGTGGGCCTTTCTGCGTTTATAAGGAGACACTTTATGTTTAAGAAGGTTGGTAAATTCCTTGCGGCTTTGGCAGCTATCCTGACGCTTGCGTATATTCTTGCGGTATACCCTCAAGTAGCACTAGTAGTAGTTGGCGCTTGTTACt                                                                                                                                                                                                   <  1:631960/301‑1 (MQ=255)`  
                                                                                                           `gCTTGTACTATTAACCCTTACGATGGCTGGGCGTGTCAAATTAGCTACATGGAGGAAACACCTGATGGCTCTTTGCGTCACCCATCGTTCGTAATGTTCCGTGGCACCGA‑GGACAACCCTCAAGAGAAAATGTAATCACACTGGCTCACCTTCGGGTGGGCCTTTCTGCGTTTATAATGAGACACTTTATGTTTAAGAAGGTTGGTAAATTCCTTGCGGCTTTGGCAGCTATCCTGACGCTTGCGTATATTCTTGCGTTATACCCTCAAGTAGCACTAGTAGTAGTTGGGGCTTTTTACtt                                                                                                                                                                                                  >  1:561532/1‑301 (MQ=255)`  
                                                                                                           `gCTTGTACTATTAACCCTTACGATGGCTGGGCGTGTCAAATTAGCTACATGGAGGAAACACCTGATGGCTCTTTGCGGCACCCATCGTTCGTAATGTTCCGTGGCACCGA‑TGACAACCCTCAAGAGAAAATGTAATCACACTGGCTCACCTTCGGGTGGGCCTTTCTGCGTTTATAAGGAGACACTTTATGTTTAAGAAGGTTGGTAAATTCCTTGCGGCTTTGGCAGCTATCCTGACGCTTGCGTATATTCTTGCGGTATACCCTCAAGtctgtctcttatacacatctcctatcccacc                                                                                                                                                                                                  >  1:56412/1‑271 (MQ=255)`  
                                                                                                              `gagatgtgtataagagacagtGGCTGGGCGTGTCAAATTAGCTACATGGAGGAAACACCTGATGGCTCTTTGCGGCACCCATCGTTCGTAATGTTCCGTGGCACCGA‑GGACAACCCTCAAGAGAAAATGTAATCACACTGGCTCACCTTCGGGTGGGCCTTTCTGCGTTTATAAGGAGACACTTTATGTTTAAGAAGGTTGGTAAATTCCTTGCGGCTTTGGCAGCTATCCTGACGCTTGCGTATATTCTTGCGGTATACCCTCAAGTAGCACTAGTAGTAGTTGGCGCTTGGTACTTAGc                                                                                                                                                                                               <  1:686970/281‑1 (MQ=255)`  
                                                                                                               `gTACTATTAACCCTTACGATGGCTGGGCGTGTCAAATTAGCTACATGGAGGAAACACCTGATGGCTCTTTGCGGCACCCATCGTTCGTAATGTTCCGTGGCACCGA‑GGACAACCCTCAAGAGAAAATGTAATCACACTGGCTCACCTTCGTGTTGGCCTTTCTGCGTTTATAAGGAGACACTTTATGTTTAAGAAGGTTGGTAAATTCCTTGCGGCTTTTGCAGCTATCCTGACGCTTGCGTATATTCTTGCGGTATACCCTCAAGTAGCACTAGTAGTAGTTGGCTCTTGTTACTTAGCg                                                                                                                                                                                              >  1:473427/1‑301 (MQ=255)`  
                                                                                                                 `aCTCTTAACCCTTACGATGGCTGGGCGTGTCAAATTAGCTACATGGAGGAAACACCTGATGGCTCGTTGCGGCACCCATCGTTCGTAATGTTCCGTGGCACCGA‑GGACAACCCTCAAGAGAAAATGTAATAACACTGGCTCACCTTAGGGTGGGCCTTTCTGCGTTTATAAGGAGACACTTTATGTTTAAGAAGGTTGGTAAATTCCTTGCGGCTTTGGCAGCTATCATGAAGCTTGCGTATATTCTTGCGGTATACCCTCAAGTAGCCATAGTAGTAGTTGGAGCTTGTTATCTAGCggg                                                                                                                                                                                            >  1:603439/1‑300 (MQ=255)`  
                                                                                                                 `aCTATTAACCCTTACGATGGCTGGGCGTGTCAAATTAGCTACATGGAGGAAACACCTGATGGCTCTTTGCGGCACCCATCGTTCGTAATGTTCCGTGGCACCGA‑GGACAACCCTCAAGAGAAAATGTAATCACACTGGCTCACCTTCGGGTGGGCCTTTCTGCGCTTATAAGGAGACACTTTATGTTTAAGAAGGTTTGTAAATTCCTTGCGGCTTTGGCAGCTATCCTGACGCTTGCGTATATTCTTGCGGTATACCCTCAAGTAGCACTAGTAGTAGTTGGCGCTTGTTACTTAGCGGc                                                                                                                                                                                            >  1:584760/1‑301 (MQ=255)`  
                                                                                                                  `cTATTAACCCTTACGATGGCTGGGCGTGTCAAATTAGCTACATGGAGGAAACACCTGATGGCTCTTTGCGGCACCCATCGTTTGTAATGTTCCGTTGCACCGA‑GGACAACCCTCAAGAGAAAATGTAATCACACTGGCTCACCTTCGGGTGGGCCTTTCTGCGTTTATAAGGAGACACTTTATGTTTAAGAAGGTTGGTAAATTCCTTGCGGCTTTGGCAGCTATCCTGACGCTTGCGTATATTCTTGCGGTATACCCTCAAGTAGCACTAGTAGTAGTTGGCGCTTGTTACTTAGCGGCa                                                                                                                                                                                           >  1:159712/1‑301 (MQ=255)`  
                                                                                                                  `cTATTAACCCTTACGATGGCTGGGCGTGTCAAATTAGCTACATGGAGGAAACACCTGATGGCTCTTTGCGGCACCCATCGTTCGTAATGTTCCGTGGCACCGA‑GGACAACCCTCAAGAGAAAATGTAATCACACTGGCTCACCTTCGGGTGGGCCTTTCTGCGTTTATAAGGAGACACTTTTTGTTTAAGTAGGTTGGTAAATTCCTTGCGGCTTTGGCAGCTATCCTGACGCTTGCGTATATTCTTGCGGTATACCCTCAAGTAGCACTTGTAGTATTTGTCGCTTTTTACTTTGCGGCa                                                                                                                                                                                           >  1:660874/1‑301 (MQ=255)`  
                                                                                                                  `cTATTAACCCTTACGATGGCTGGGCGTGTCAAATTAGCTACATGGAGGAAACACCTGATGGCTCTTTGCGGCACCCATCGTTCGTAATGTTCCGTGGCACCGA‑GGACAACCCTCAAGAGAAAATGTAATCACACTGGCTCACCTTAGGGTGGGCCTTTCTGCGTTTATAAGGAGACACTTTATGTTTAAGAAGGTTGGTAAATTCCTTGCAGCTTTGGCAGCTATCATGACGCTTGCGTATATTCTTGCGTGATACCCTCAAGTAGCACTCGTAGTAGTTGGCGCTTGTTACTTagcagcc                                                                                                                                                                                           >  1:186841/1‑300 (MQ=255)`  
                                                                                                                   `tATTAACCCTTACGATGGCTGGGCGTGTCAAATTAGCTACATGGAGGAAACACCTGATGGCTCTTTGCGGCACCCATCGTTCGTAATGTTCCGTGGCACCGA‑GGACAACCCTCAAGAGAAAATGTAATCACACTGGCTCACCTTCGGATGGGCCTTTCTGCGTTTATAAGGAGACACTTTATGTTTAAGAAGGTTGGTAAATTCCTTGCGGCTTTGGCAGCTATCCTGACGCTTGCGTATATTCTTGCGGTATACCCTCAAGTAGCACTAGTAGTAGCTGTCTCttatacacatctccgag                                                                                                                                                                                          >  1:353984/1‑286 (MQ=255)`  
                                                                                                                     `ttAACCATTACAATGGCTGGGCGTGTCAAATTAGCTACATGGAGGAAACACCTGATGGCTCTTTGCGGCACCCATCGTTCGTAATGTTCCGTGGCACCGA‑GGACAACCCTCAAGAGAAAATGTAATCACACTGGCTCACCTTCGGGTGGGCCTTTCTGCGTTTATAAGGAGACACTTTATGTTTAAGAAGGTTGGTAAATTCCTTGCGGCTTTGGCAGCTATCCTGACGCTTGCGTATATTCTTGCGGTATACCCTCAAGTAGCACTAGTAGTAGTTGGCGCTTGTTACTTAGCGGCAgtg                                                                                                                                                                                        <  1:800763/301‑1 (MQ=255)`  
                                                                                                                         `cccTTACGATGGCTGTGCGTGTCAAATTAGCTACATGGAGGAAACACCTGATGGCTCTTTGCGGCACCCATCGTTCGTAATGTTCCGTGGTACCGA‑GGACAACCCTCAAGAGAAAATGTAATCACACTGGCTCACCTTCGGGTGGGCCTTTCTGCGTTTATAAGGAGACACTTTATGTTTAAGAAGGTTGGTAAATTCCTTGCGGCTTTGGCAGCTATCCTGACGCTTGCGTATATTCTTGCGGTATACCCTCAAGTAGCACTAGTAGTCGTTGGCGCTTGTTACTTAGCGGCAGtgtgtg                                                                                                                                                                                    >  1:665910/1‑301 (MQ=255)`  
                                                                                                                         `cccTTACGATGGCTGGGCGTGTCAAATTAGCTACATGGAGGAAACACCTGATGGATCTTTGCGGCACCCATAGTTCGTAATGTTCCGTGGCACCGA‑GGACAACCCTCAAGAGAAAATGTAATCACACTGGCTCACCTTCGGGTGGGCCTTTCTGCGTTTATAAGGAGACACTTTATGTTTAAGAAGGTTGGTAAATTCCTTGCGGCTTTGGCAGCTATCATGACGCTTGCGTATATTCTTGCGGTATACCCTCAAGTAGCACTAGTAGTAGTTGGCGCTTGTTACTTAGCGGCAGtgtgtg                                                                                                                                                                                    >  1:253856/1‑301 (MQ=255)`  
                                                                                                                           `cTTACGATGGCTGGGCGTGTCAAATTAGCTACATGGAGGAAACACCTGATGGCTCTTTGCGGCACCCATCGTTCGTAATGTTCCGTGGCACCGA‑GGACAACCCTCAAGAGAAAATGTAATCACACTGGCTCACCTTCGGGTGGGCCTTTCTGCGTTTATAAGGAGACACTTTATGTTTAAGAAGGTTGGTAAATTCCTTGCGGCTTTGGCAGCTATCCTGACGCTTGCGTATATTCTTGCGGTATACCCTCAAGTAGCACTAGTAGTAGTTGGCGCTTGTTACTTAGCGGCAGTGTGTGCt                                                                                                                                                                                  <  1:437355/301‑1 (MQ=255)`  
                                                                                                                             `tACGATGGCTGGGCGTGTCAAATTAGCTACATGGAGGAAACACCTGATGTCTCTTTGCGGCACCCATCGTTCGTAATGTTCCGTGGCACCGA‑GGACAACCCTCAAGAGAAAATGTAATCACACTGGCTCACCTTCGGGTGGGCCTTTCTGCGTTTATAAGGAGACACTTTATGTTTAAGAAGGTTGGTAAATTCCTTGCGGCTTTGGCAGCTATCCTGACGCTTGCGTATACTCTTGCGGTATACCCTCAAGTAGCACTAGTAGTAGTTGGCGCTTGTTACTTAGCGGCAGTGTGTGCttt                                                                                                                                                                                >  1:444174/1‑300 (MQ=255)`  
                                                                                                                             `tACGATGGCTGGGCGTGTCAAATTAGCTACATGGAGGAAACACCTGACGGCTCTTTGCGGCACCCATAGTTCGTAATGTTCCGTGGCACCGA‑GGACAACCCTCAAGAGAAAATGTAATCACACTGGCTCACCTTCGGGTGGGCCTTTCTGCGTTTATAAGGAGACACTTTATGTTTAAGAAGGTTGGTAAATTCCTTGCGGCTTTGGCAGCTATCCTGACGCTTGCGTATATTCTTGCGGTATACCCTCAAGTAGCACTAGTAGTAGTTGGCGCTTGTTACTTAGCGGCAGTGTGTGCTTg                                                                                                                                                                                >  1:450307/1‑301 (MQ=255)`  
                                                                                                                                    `gCTGGGCGTGTCAAATTAGCTACATGGAGGAAACACCTGATGGCTCTTTGCGGCACCCATCGTTCGTAATGTTCCGTGGCACCTA‑GGACAACCCTCAAGAGAAAATGTAATCACACTGGCTCACCTTCGGGTGGGCCTTTCTGCGTTTATAAGGAGACACTTTATGTTTAAGAAGGTTGGTAAATTCCTTGCGGCTTTGGTAGCTATCCTGACGCTTGCGTATATTCTTGCGGTATACCCTCAAGTAGCACTAGTAGTAGTTGGCGCTTGTTACTTAGCGGCAGTGTGTGCTTGCGTGTgg                                                                                                                                                                         <  1:311921/301‑1 (MQ=255)`  
                                                                                                                                      `tGGGCGTGTCAAATTAGCTACATGGAGGAAACACCTGATGGCTCTTTGCGGCACCCATCGTTCGTAATGTTCCGTGGCACCGA‑GGACAACCCTCAAGAGAAAATGTAATCACACTGGCTCACCTTCGTGTGGGCCTTTCTGCGATTATAAGGAGACACTTTATGTTTAAGAAGGTTGGTAAATTCCTTGCGGCTTTTGCAGCTATCCTGACGCTTGCGTATATTCTTGTGGTATACCCTCAAGTAGCACTAGTAGTAGTTGGCGCTTGTTACTTAGCGGCAGTGTGTGCTTGCGTGTGGAg                                                                                                                                                                       <  1:101395/301‑1 (MQ=255)`  
                                                                                                                                       `gggCGTGTCAAATTAGCTACATGGAGGAAACACCTGATGGCTCTTTGCGGCACCCATCGTTCGTAATGTTCCGTGGCACCGA‑GGACAACCCTCAAGAGAAAATGTAATCACACTGGCTCACCTTCGGGTGGGCCTTTCTGCGTTTATAAGGAGACACTTTATGTTTAAGAAGGTTGGTACATTCCTTGCGGCTTTGGCAGCTATCCTGACGCTTGCGTATATTCTTGCGGTATACCCTCAAGTAGCACTAGTAGTAGTTGGCGCTTGTTACTTAGCGGCAGTGTGTGCTTGCgtgttgggt                                                                                                                                                                      >  1:409823/1‑296 (MQ=255)`  
                                                                                                                                            `tgtCAAATTAGCTACATGGAGGAAACACCTGATGGCTCTTTGCGGCACCCATCGTTCGTAATGTTCCGTGGCACCGA‑GGACAACCCTCAAGAGAAAATGTAATCACACTGGCTCACCTTCGGGTGGTCCTTTCTGCGTTTATAAGGAGACACTTTATGTTTAAGAAGGTTGGTAAATTCCTTGCGGCTTTGGCAGCTATCCTGACGCTTGCGTATATTCTTGCGGTATACCCTCAAGTAGCACTAGTAGTAGTTGGCGCTTGTTACTTAGCGGCAGTGTGTGCTTGCGTGTGGAGTATAGt                                                                                                                                                                 <  1:815705/301‑1 (MQ=255)`  
                                                                                                                                            `gttCAAATTAGCTACATGGATGAAACACCTGATGGCTCTTTGCGGCACCCATCGTTCGTAATGTTCCGTGGCACCGA‑GGACAACCCTCAAGAGAAAATGTAATCACACTGGCTCACATTCGGGTGGGCCTTTCTGCGTTTATAAGGAGACACTTTATGTTTAAGAAGGTTGGTAAATTCCTTGCGGCTTTGGCAGCTATCATGACGCTTGCGTATATTCTTGCGGTATACCCTCAAGTAGCACTAGTAGTAGTTGGCGCTTGTTACTTAGCGGCAGTGTGTGCTTGCGTGTGGAGTATAGt                                                                                                                                                                 <  1:762802/299‑1 (MQ=255)`  
                                                                                                                                              `gtgtataagagaCAGGGAGGAAACACATGATGGATCTTTGCGGCACCCATCGTTCGTAATGTTCCGTGGCACCGA‑GGACAACCCTCAAGAGAAAATGTAATCACACTGGCTCACCTTCGGGTGGGCCTTTCTGCGTTTATAAGGAGACACTTTATGTTTAAGAAGGTTGGTAAATTCCTTGCGGCTTTGGCAGCTATCCTGACGCTTGCGTATATTCTTGCGGTATACCATCAAGTAGCACTAGTAGTAGTTGGCGCTTGTTACTTAGCGGCAGTGTGTGCTTGCGTGTGGAGTATAGTTa                                                                                                                                                               <  1:371589/290‑1 (MQ=255)`  
                                                                                                                                                  `aTTAGCTACATGGAGGAAACACCTGATGGCTCGTTGCGGCACCCATCGTTCGTAATGTTCCGTGGCACCGA‑TGACAACCCTCAAGAGAAAATGTAATCACACTGGCTCACCTTCGGGTGGGCCTTTCTGCGTTTATAAGGAGACACTTTATGTTCAAGAAGGTTGGTAAATTCCTTGCGGCTTTGGCAGCTATCCTGACGCTTGCGTATATTCTTGCGGTATACCCTCAAGTAGCACTAGTAGTAGTTGGCGCTTGTTACTTAGCGGCAGTGTGTGCTTGCGTGTGGAGTATAGTTAACTg                                                                                                                                                           <  1:301662/301‑1 (MQ=255)`  
                                                                                                                                                         `aCATGGAGGAAACACCTGATTGCTCTTTGCGGCACCCATCGTTCGTAATGTTCCGTGGCACCGA‑GGACAACCCTCAAGAGAAAATGTAATCACACTGGCTCACCTTCGGGTGGGCCTTTCTGCGTTTATAAGGAGACACTTTATGTTTAAGAAGGTTGGTAAATTCCTTGCGGCTTTGGCAGCTATCCTGACGCTTGCGTATATTCTTGCGGTATACCCTCAAGTAGCACTAGTAGTAGTTGGCGCTTGTTACTTAGCGGCAGTGTGTGCTTGCGTGTGGAGTATAGTTAACTGGTAATAc                                                                                                                                                    <  1:106629/301‑1 (MQ=255)`  
                                                                                                                                                          `cATGGAGGAAACACCTGATGGCTCTTTGCGGCACCCATCGTTCGTAATGTTCCGTGGCACCGA‑GGACAACCCTCAAGAGAAAATGTAATCACACTGGCTCACCTTCGGGTGGGCCTTTCTGCGTTTATAAGGAGACACTTTATGTTTAAGAAGGTTGGTAAATTCCTTGCGGCTTTGGCAGCTATCCTGACGCTTGCGTATATTCTTGCGGTATACCCTCAAGTAGCACTAGTAGTAGTTGGCGCTTGTTACTTAGCGGCAGTGTGTGCTTGCGTGCGGAGTATAGTTAACTGGTAATACg                                                                                                                                                   <  1:214173/301‑1 (MQ=255)`  
                                                                                                                                                            `gggaggaAACACCTGATGGCTCTTTGCGGCACCCATCGTTCGTAATGTTCCGTGGCACCGA‑GGACAACCCTCAAGAGAAAATGTAATCACACTGGCTCACCTTCGGGTGGGCCTTTCTGCGTTTATAAGGAGACACTTTATGTTTAAGAAGGTTGGTAAATTCCTTGCGGCTTTGTCAGCTATCCTGACGCTTGCGTATATTCTTGCGGTATACCCTCAAGTAGCACTAGTAGTAGTTGGCGCTTGTTACTTAGCGGCAGTGTGTGCTTGCGTGTGGAGTATAGTTAACTGGTAATACGAc                                                                                                                                                 <  1:694199/300‑1 (MQ=255)`  
                                                                                                                                                             `ggaggaAACACCTGATGGCTCTTTGCGGCACCCATCGTTCGTAATGTTCCGTGGCACCGA‑GGACAACCCTCAAGAGAAAATGTAATCACACTGGCTCACCTTCGGGTGGGCCTTTCTGCGTTTATAAGGAGACACTTTATGTTTAAGAAGGTTGGTAAATTCCTTGCGGCTTTGGCAGCTATCCTGACGCTTGCGTATATTCTTGCGGTATACCCTCAAGTAGCACTAGTAGTAGTTGGCGCTTGTTACTTAGCGGCAGTGTGTGCTTGCGTGTGGAGgatctgtctcttatacacatctc                                                                                                                                                >  1:340143/1‑281 (MQ=255)`  
                                                                                                                                                              `gaggaAACACCTGATGTCTCTTTGCGGCACCCATCGTTCGTAATGTTCCGTGGCACCGA‑GGACAACCCTCAAGAGAAAATGTAATCACACTGGCTCACCTTCGGGTGGGCCTTTCTGCGTTTATAAGGAGACACTTTATGTTTAAGAAGGTTGGTAAATTCCTTGCGGCTTTGGCAGCTATCCTGACGCTTGCGTATATTCTTGCGGTATACCATCAAGTAGCACTAGTAGTAGTTGGCGCTTGTTACTTAGCGGCAGTGTGTGCTTGCGTGTGGAGTATAGTTAACTGGTAATACGACTc                                                                                                                                               >  1:165491/1‑301 (MQ=255)`  
                                                                                                                                                              `gaggaAACACCTGATGGCTCTTTGCGGCACCCATCGTTCGTAATGTTCCGTGGCACCGA‑GGACAACCCTCAAGAGAAAATGTAATCACACTGGCTCACCTTCGGGTGGGCCTTTCTGCGTTTATAAGGAGACACTTTATGTTTAAGAAGGTTGGTAAATTCCTTGCGGCTTTGGCAGCTATCCTGACGCTTGCGTATATTCTTGCGGTATACCCTCAAGTAGCACTAGTAGTAGTTGGCGCTTGTTACTTAGCGGCAGTGTGTGCTTGCGTGTGGAgctgtctcttatacacatctccgag                                                                                                                                               >  1:824461/1‑277 (MQ=255)`  
                                                                                                                                                                `ggaAACACCTGATGGCTCTTTGCGGCACCCATCGTTCGTAATGTTCCGTGGCACCGA‑GGACAACCCTCAAGAGAAAATGTAATCACACTGGCTCACCTTCGGGTGGGCCTTTCTGCGTTTATAAGGAGACACTTTATGTTTAAGAGGGTTGGTAAATTCCTTGCGGCTTTGGCAGCTATCCTGACGCTTGCGTATATTCTTGCGGTATACCCTCAAGTAGCACTAGTAGTAGTTGGCGCTTGTTACTTATCGGCAGTGTGTGCTTGCGTGTGGCGTATAGTTAACTGGTAATACGACttag                                                                                                                                             >  1:17323/1‑298 (MQ=255)`  
                                                                                                                                                                      `gtgggctcggagatgtgtataagagacagtcgtAATGTTCCGTGGCACCGA‑GGACAACCCTCAAGAGAAAATGTAATCACACTGGCTCACCTTCGGGTGGGCCTTTCTGCGTTTATAAGGAGACACTTTATGTTTAAGAAGGTTGGTAAATTCCTTGCGGCTTTGGCAGCTATCCTGACGCTTGCGTATATTCTTGCGGTATACCCTCAAGTAGCACTAGTAGTAGTTGGCGCTTGTTACTTAGCGGCAGTGTGTGCTTGCGTGTGGAGTATAGTTAACTGGTAATACGACTCACTAAagg                                                                                                                                       <  1:127404/272‑1 (MQ=255)`  
                                                                                                                                                                      `ataagagacagctTTGTGGCACCCATCGTTCGTAATGTTCCGTGGCACCGA‑GGACAACCCTCAAGAGAAAATGTAATCACACTGGCTCACCTTCGGGTGGGCCTTTCTGCGTTTATAAGGAGACACTTTATGTTTAAGAAGGTTGGTAAATTCCTTGCGGCTTTGGCAGCTATCCTGACGCTTGCGTATATTCTTGCGGTATACCCTCAAGTAGCACTAGTAGTAGTTGGCGCTTGTTACTTAGCGGCAGTGTGTGCTTGCGTGTGGAGTATAGTTAACTGGTAATACGACTCACTAAagg                                                                                                                                       <  1:265832/290‑1 (MQ=255)`  
                                                                                                                                                                      `acCTGGTGGCTTTTTGGGTGATCCCTCGTTCTGTATGTTCCGGTGCACCCATTGCCACCCCTC‑AGAGTAAATTGTATCACACTTGCTCACCCTTGGGTGGGCCTTTCTGCGTTTATAAGGAGACACTTTATGTTTAAGAAGGTTGGTAAATTCCTTGCGGCTTTGGCAGCTATCCTGACGCTTGCGTATATTCTTGCGGTATACCCTCAAGTAGCACTAGTAGTAGTTGGCGCTTGTTACTTAGCGGCAGTGTGTGCTTGCGTGTGGAGTATAGTTAACTGGTAATACGACTCACTAAagg                                                                                                                                       <  1:792772/301‑1 (MQ=255)`  
                                                                                                                                                                              `tctctTTGCGGCACCCATCGTTCGTAATGTTCCGTGGCACCGA‑GGACAACCCTCAAGAGAAAATGTAATCACACTGGCTCACCTTCGGGTGGGCCTTTATGCGTTTATAATGAGACACTTTATGTTTAAGAACGTTGGTAAATTCCTTGCGGCTTTGGCAGCTATCCTGACGCTTGCGTATATTCTTGCGGTATACCCTCAAGTAGCACTAGTAGCAGTTGGCGCTTGTTACTTAGCGGCAGTGTGTGCTTTCGTGTGGAGTATAGTTAACTGGTAATACGACTCACTAAAGGAGGTacac                                                                                                                               <  1:54536/300‑1 (MQ=255)`  
                                                                                                                                                                                            `ccATCGTTCGTAATGTTCAGTGGCACCGA‑GGACAACCCTCAAGAGAAAATGTAATCACACTGGCTCACCTTCGGGTGGGCCTTTCTGCGTTTATAAGGAGACACTTTATGTTTAAGAAGGTTGGTAAATTACTTGCGGCTTTGGCAGCTATCCTGACGCTTGCGTATATTCTTGCGGTATACCCTCAAGTAGCACTAGTAGTAGTTGGCGCTTGTTACTTAGCGGCAGTGTGTGCTTGCGTGTGGAGTATAGTTAACTGGTAATACGACTCACTAAAGGAGGTACACACCATGATGTACtt                                                                                                                 <  1:241492/301‑1 (MQ=255)`  
                                                                                                                                                                                               `tcgttcgtAATGTTCCGTGGCACCGA‑GGACAACCCTCAAGAGAAAATGTAATCACACTGGCTCACCTTCGGGTGGGCCTTTCTGCGTTTATAAGGAGACACTTTATGTTTAAGAAGGTTGGTAAATTCCTTGCGGCTTTGGCAGCTATCCTGACGCTTGCGTATATTCTTGCGGTATACCCTCAAGTAGCACTAGTAGTAGTTGGCGCTTGTTACTTAGCGGCAGTGTGTGCTTGCGTGTGGAGTATAGTTAACTGGTAATACGACTCACTAAAGGAGGTACACACCATGATGTACTTAAt                                                                                                              <  1:642036/301‑1 (MQ=255)`  
                                                                                                                                                                                                 `gttcgtAATGTTCCGTGGCACCGA‑GGACAACCCTCAAGAGAAAATGTAATCACACTGGCTCACCTTCGGGTGGGCCTTTCTGCGTTTATAAGGAGACACTTTATGTTTAAGAAGGTTGGTAAATTCCTTGCGGCTTTGGCAGCTATCCTGACGCTTGCGTATATTCTTGCGGTATACCCTCAAGTAGCACTAGTAGTAGTTGGCGCTTGTTACTTAGCGGCAGTGTGTGCTTGCGTGTGGAGTATAGTTAACTGGTAATACGACTCACTAAAGGAGGTACACACCATGATGTACTTAATGc                                                                                                            <  1:720593/301‑1 (MQ=255)`  
                                                                                                                                                                                                    `cgtAATGTTCCGTGGCACCGA‑GGACAACCCTCAAGAGAAAATGTAATCACACTGGCTCACCTTCGGGTGGGCCTTTCTGCGTTTATAAGGAGACACTTTATGTTTAAGAAGGTTGGTAAATTCCTTGCGGCTTTGGCAGCTATCCTGACGCTTGCGTATATTCTTGCGGTATACCCTCAAGTAGCACTAGTAGTAGTTGGAGCTTGTTACTTAGCGGCAGTGTGTGCTTGCGTGTGGAGTATAGTTAACTGGTAATACGACTCACTAAAGGAGGTACACACCATGATGTACTTAATGCCAt                                                                                                         <  1:595878/301‑1 (MQ=255)`  
                                                                                                                                                                                                     `gtAATGTTCCGTGGCACCGA‑GGACAACCCTCAATAGAAAATGTAATCACACTGGCTCACCTTCGGGTGGGCCTTTCTGCGTTTATAAGGAGACACTTTATGTTTAAGAAGGTTGGTAAATTCCTTGCGGCTTTGGCAGCTATCCTGACGCTTGCGTATATTCTTGCGGTATACCCTCAAGTAGCACTAGTAGTAGTTGGCGCTTGTTACTTAGCGGCAGTGTGTGCTTGCGTGTGGAGTATAGTTAACTGGTAATACGACTCACTAAaggagacactatatgtttcgacttcagtacaaca                                                                                                        >  1:182594/1‑272 (MQ=255)`  
                                                                                                                                                                                                         `tGTTCCATGGTACCGA‑GGACAACCCTCAAGAGAAAATGTAATCACACTGGCTCACCTTCGGGTGGGCCTTTCTGCTTTTATAAGGAGACACTTTATGTTTAAGAAGGTTGGTAAATTCCTTGCGGCTTTGGCAGCTATCCTTACGCTTGCGTATATTCTTGCGGTATACCCTCAAGTAGCACTAGTAGTAGTTGGCGCTTGTTACTTAGCGGCAGTGTGTGCTTGCGTGTGGAGTATAGTTAACTGGGAATACGACTCACTAAAGTAGGTACACACCATGAGGGAAGTAATGCAAGTACTc                                                                                                    <  1:807277/301‑1 (MQ=255)`  
                                                                                                                                                                                                                  `gggctcggagatgtgtataagagacagaTGTAATCACACTGGCTCACCTTCGGGTGGGCCTTTCTGCGTTTATAAGGAGACACTTTATGTTTAAGAAGGTTGGTAAATTCCTTGCAGCTTTGGCAGCTATCCTGACGCTTGCGTATATTCTTGCGGTATACCCTCAAGTAGCACTAGTAGTAGTTGGCGATTGTTACTTAGCGGCAGTGTGTGCTTGCGTGTGGAGTATAGTCAACTGGTAATACGACTCACTAAAGGAGGTACACACCATGATGTACTTAATGCCATTACTcatcatcat                                                                                            <  1:262682/274‑1 (MQ=255)`  
                                                                                                                                                                                                                  `gCACCTA‑GGACAACCCTCAAGAGAAAATGTAATCACACTGGCTCACCTTCGGGTGGGCCTTTCTGCGTTTATAAGGAGACACTTTATGTTTAAGAAGGTTGGTAAATTCCTTGCGGCTTTGGCAGCTATCCTGACGCTTGCGTATATTCTTGCGGTATACCCTCAAGTAGCACTAGTAGTAGTTGGCGCTTGTTACTTAGCGGCAGTGTGTGCTTGCGTGTGGAGTATAGTTAACTGGTAATACGACTCACTAAAGGAGGTACACACCATGACGTACTTAATGCCATTACTCATCGTCAtt                                                                                           >  1:22732/1‑301 (MQ=255)`  
                                                                                                                                                                                                                  `gCACCGA‑GGACAACCCTCAAGAGAAAATGTAATCACACTGGCTCACCTTCGGGTGGGCCTTTCTGCGTTTATAAGGAGACACTTTATGTTTAAGAAGGTTGGTAAATTCCTTGCGGCTTTGGCAGCTATCCTGACGCTTGCGTATATTCTTGCGGTATACCCTCAAGTAGCACTAGTAGTAGTTGGCGCTTGTTACTTAGCGGCAGTGTGTGCTTGCGTGTGGAGTATAGTTAACTGGTAATACGACTCACTAAAGGAGGTACACACCatgatctgtctcttatacacatctccgagccca                                                                                           >  1:571907/1‑273 (MQ=255)`  
                                                                                                                                                                                                                  `gCACCGA‑GGACAACCCTCAAGAGAAAATGTAATCACACTGGCTCACCTTCGGGTGGGCCTTTCTGCGTTTATAAGGAGACACTTTATGTTTAAGAAGGTTGGTAAATTCCTTGCGGCTTTGGCAGCTATCCTGACGCTTGCGTATATTCTTGCGGTATACCCTCAAGTAGCACTAGTAGTAGTTGGCGCTTGTTACTTAGCGGCAGTGTGTGCTTGCGTGTGGAGTATAGTTAACTGGTAATACGACTCACTAAAGGAGGTACACACCATGATGTAcctgtctcttatacacatctccgcg                                                                                           >  1:154934/1‑277 (MQ=255)`  
                                                                                                                                                                                                                    `aCCGA‑GTACAACCCTCAAGAGAAAATGTAATCACACTGGCTCACCTTCGGGTGGGCCTTTCTGCGTTTATAAGGAGACACTTTATGTTTAAGAAGGTTGGTAAATTCCTTGCGGCTTTGGCAGCCATCCTTACTCTTGCGTATATTCTTGCGGTATACCCTCAAGTAGCACTAGTAGTAGTTGGCGCTTGTTACTTAGCGGCAGTGTGTGCTTGCGTGTGGAGTATAGTTAACTGGTAATACGACTCACTAAAGGAGGTACACACCATGATGTACTTAATGCCATTACTCATCGTCATTGt                                                                                         >  1:344859/1‑301 (MQ=255)`  
                                                                                                                                                                                                                     `ccGA‑GGACAACCCTCAAGAGAAAATGTAATCACACTGGCTCACCTTCGGGTGGGCCTTTCTGCGTTTATAAGGAGACACTTTATGTTTAAGAAGGTTGGTAAATTCCTTGCTGCTTTGGCAGCTATCCTGACGCTTGCGTATATTCTTGCGGTATACCCTCAAGTAGCACTAGTAGTAGTTGGCGCTTGTTACTTAGCGGCAGTGTGTGCTTGCGTGTGCAGTATAGTTAACTGGTAATACGACTCACTAAAGGAGGTACACACCATGATGTACTTAATGCCATTACTCATCGTCATTGTa                                                                                        >  1:197593/1‑301 (MQ=255)`  
                                                                                                                                                                                                                      `cGA‑GGACAACCCTCAAGAGAAAATGTAATCACACTGGCTCACCTTCGGGTGGGCCTTTCTGAGTTTATAAGGAGACACTTTATGTTTAAGAAGGTTGGTAAATTCCTTGCGGCTTTGGCAGCTATCCTGACGCTTGCGTATATTCTTGCGGTATACCCTCAAGTAGCACTAGTAGTAGTTGGCGCTTGTTACTTAGCGGCAGTGTGTGCTTGCGTGTAGAGTATAGTTAACTGGTAATACGATTCACTAAAGGAGGTACACACCATGATGTACTTAATGCCATCACTCATCGTCATTGTAg                                                                                       <  1:141553/301‑1 (MQ=255)`  
                                                                                                                                                                                                                          `ggACAACCCTCAAGAGAAAATGTAATCACACTGGCTCACCTTCGGGTGGGCCTTTCTGCGTTTATAAGGAGACACTTTATGTTTAAGAAGGTTGGTAAATTCCTTGCGGCTTTGGCAGCTATCCTGACGCTTGCGTATATTCTTGCGGTATACCCTCAAGTAGCACTAGTAGTAGTTGGCGCTTGTTACTTAGCGGCAGTGTGTGCTTGCGTGTGGAGTATAGTTAACTGGTAATACGACTCACTAAAGGAGGTACACACCATGATGTACTTAATGCCATTACTCATCGTCATTGTAGGAt                                                                                    >  1:705434/1‑301 (MQ=255)`  
                                                                                                                                                                                                                                             `aagagacagcacTGGCTCACCTTCGGGTGGGCCTTTCTGCGTTTACAAGGAGACACTTTATGTTTAAGAAGGTTGGTAAATTCCTTGCGGCTTTGGCAGCTATCCTGACGCTTGCGTATATTCTTGCGGTATACCCTCAAGTAGCACTAGTAGTAGTTGGCGCTTGTTACTTAGCGGCAGTGTATGCTTGCGTGTGGAGTATAGTTAACTGGTAATATGACTCACTAAAGGATGTACACACCATGATGTACTTAATGCCATTACTCATCGTCATTGTAGGATGCCTTGCGCTCCACTGTAg                                                                 <  1:272943/292‑1 (MQ=255)`  
                                                                                                                                                                                                                                             `aagTAATCACACTGGCTCACCTTCGGGTGGGCCTTTCTGCGTTTATAAGGAGACACTTTATGTTTAAGAAGGTGGGTAAATTCCTTGCGGCTTTGGCAGCTATCCTGACGCTTGCGTATATTCTTGCAGTATACCCTCAAGTAGCACTAGTAGTAGTTGGCGCTTGTTACTTAGCGGCAGTGTGTGCTTGCGTGTGGAGTATAGTTAACTGGTAATACGACTCACTAAAGGAGGTACACACCATGATGTACTTAATGCCATTACTCATCGTCATTGTAGGATGCCTTGCGCTCCACTGTAg                                                                 <  1:45629/299‑1 (MQ=255)`  
                                                                                                                                                                                                                                             `aTGTAATCACACTGGCTCACCTTCGGGTGGGCCGTTCTGCGTTTATAAGGAGACACTTTATGTTTAAGAAGGTTGGTAAATTCCTTGCGGCTTTGGCAGCTATCCTGACGCTTGCGTATATTCTTGCGGTATACCCTCAAGTAGCACTAGTAGTAGTTGGCGCTTGTTACTTAGCGGCAGTGTGTGCTTGCGTGTGGAGTATAGTTAACTGGTAATACGACTCACTAAAGGAGGTACACACCATGATGTACTTAATGCCATTACTCATCGTCATTGTAGGATGCCTTGCGCTCCACTGTAg                                                                 <  1:418915/301‑1 (MQ=255)`  
                                                                                                                                                                                                                                               `gTAATCACACTGGCTCACCTTCGGGTGGGCCTTTCTGCGTTTATAAGGAGACACTTTATGTTTAAGAAGGTTGTTAAATTCCTTGCGGCTTTGGCAGCTCTCCTGTCGCTTGCGTTTATTCTTGCGGTCTACCCTCAAGTAGCACTATTATTAGTTGGCGCTTGTTACTTAGCGTCAGTTTGTGCTTTCGTGTGGAGTATAGTTAACTGGTAATACGACTCACTAAAGGAGGTACACACCATGATGTACTTAATGCCATTACTCATCGTCATTGTAGGATGCCTTGCGTTCCACTGTatct                                                               >  1:234568/1‑298 (MQ=255)`  
                                                                                                                                                                                                                                                 `aaTCACACTGGCTCACCTTCGGGTGGGCCTTTCTGCGTTTATAAGGAGACACTTTATGTTTAAGAAGGTTGGTAAATTCCTTGCGGCTTTGGCATCTATCCTGACGCTTGCGTATATTCTTGCGGTATACCCTCAAGTAGCACTAGTAGTAGTTGGCGCTTGTTACTTAGCGGTAGTGTGTGCTTGCGTGTGGAGTATAGTTAACTGGTAATACGACTCACTAAAGGAGGTACACACCATGCTGTACTTAATGCCATTACTCATCGTCATTGTAGGATGCCTTGCGCTCCACTGTAGCgat                                                             >  1:627699/1‑301 (MQ=255)`  
                                                                                                                                                                                                                                                     `acacTGGCTCACCTTCGGGTGGGCCTTTCTGCGTTTATAAGGAGACACTTTATGTTTAAGAAGGTTGGTAAATTCCTTGCGGCTTTGGCAGCTATCCTGACGCTTGCGTATATTCTTGCGGTATACCCTCAAGTAGCACTAGTAGTAGTTGGCGCTTGTTACTTAGCGGAAGTGTGTGCTTGCGTGTGGAGTATAGTTAACTGGTAATACGACTCACTAAAGGAGGTAAACACCATGATGTACTTAATGCCATTACTCATCGTCATTGTAgctgtctcttatacacatctccgagcccacg                                                         >  1:677218/1‑271 (MQ=255)`  
                                                                                                                                                                                                                                                          `ggCTCACCTTCGGGTGGGCCTTTCTGCGTTTATAAGGAGACACTTTATGTTTAAGAAGGTTGGTAAATTCCTTGCGGCTTTGGCAGCTATCCTGACGCTTGCGTATATTCTTGCGGTATACCCTCAAGTAGCACTAGTAGTAGTTGGCGCTTGTTACTTAGCGGCAGTGTGTGCTTGCGTGTGGAGTATAGTTAACTGGTAATACGACTCACTAAAGGAGGTACACACCATGATGTACTTAATGCCATTACTCATCGTCATTGTAGGATGCCTTGCGCTCCACTGTAGCGATGATGATATg                                                    >  1:204183/1‑301 (MQ=255)`  
                                                                                                                                                                                                                                                                        `tGGGCCTTTCTGCGTTTATAAGGAGACACTTTATGTTTAAGAAGGTTGGTAAATTCCTTGTGGCTTTGGCAGCTATCCTGACGCTTGCGTATATTCTTGCGGTATACCCTCAAGTAGCACTAGTAGTAGTTGGCGCTTGTTACTTAGCGGCAGTGTGTGCTTGCGTGTGGAGTATAGTTAACTGGTAATACGACTCACTAAAGGAGGTACACACCATGATGTACTTAATGCCATTACTCATCGTCATTGTAGGATGCCTTGCGCTCCACTGTAGCGATGATGATATGCCAGATGGTCACGc                                      <  1:57634/301‑1 (MQ=255)`  
                                                                                                                                                                                                                                                                              `tttCTGCGTTTATAAGGAGACACTTTATGTTTAAGAAGGTTAGTAAATTCCTTGCGGCTTTGGCAGCTATCCTGACGCTTGCGTATATTCTTGCGGTATACCATCAAGTAGCACTAGTAGTAGTTGGCGCTTGTTACTTAGCGGCAGTGTGTGCTTGCGTGTGGAGTATAGTTAACTGGTAATACGACTCACTAAAGGAGGTACACACCATGATGTACTTAATGCCATTACTCATCGTCATTGTAGGATGCCTTGCGCTCCACTGTAGCGATGATGATATGCCAGATTGTCACGCTTAATa                                <  1:760976/301‑1 (MQ=255)`  
                                                                                                                                                                                                                                                                               `ttCTGCGTTTATAAGGAGACACTTTATGTTTAAGAAGGTTGGTAAATTCCTTGCGGCTTTGGCAGCTATCCTGACGCTTGCGTATATTCTTGTGGTATACCCTCAAGTAGCACTAGTAGTAGTTGGCGATTGTTACTTAGCGGCAGTGTGTGCTTGCGTGTGGAGTATAGTTAACTGGTAATACGACTCACTAAAGGAGGTACACACCATGATGTACTTAATGCCATTACTCATCGTCATTGTAGGATGCCTTGCGCTCCACTGTAGCGATGATGATATGCCAGATGGTCACGCTTAATAc                               <  1:731453/301‑1 (MQ=255)`  
                                                                                                                                                                                                                                                                                            `tgtgtataagagacagttAAGAAGGTTGGTAAATTCCTTGCGGCTTTGGCAGCTATCCTGACGCTTGCGTATATTCTTGCGGTATACCCTCAAGTAGCACTAGTAGTAGTTGGCGCTTGTTACTTAGCGGCAGTGTGTGCTTGCGTGTGGAGTATAGTTAACTGGTAATACGACTCACTAAAGGAGGTACACACCATGATGTACTTAATGCCATTACTCATCGTCATTGTAGGATGCCTTGCGCTCCACTGTAGCGATGATGATATGCCAGATGGTCACGCTTAATACGACTCACTAAAgg                  <  1:249531/285‑1 (MQ=255)`  
                                                                                                                                                                                                                                                                                              `gagaCACTTTATGTTTAAGAAGGTTGGTAAATTCCTTGCGGCTTTGGCAGCTATCCTGACGCTTGCGTATATTCTTGCGGTATACCCTCAAGTAGCACTAGTAGTAGTTGGCGCTTGTTACTTAGCGGCAGTGTGTGCTTGCGTGTGGAGTATAGTTAACTGGTAATACGACTCACTAAAGGAGGTACACACCATGATGTACTTAATGCCATTACTCATCGTCATTGTAGGATGCCTTGCGCTACACTGTAGCGATGATGATATGCCAGATGGTCACGCTTAATACGACTCACTAAAggcg                >  1:518134/1‑299 (MQ=255)`  
                                                                                                                                                                                                                                                                                                  `cacTTTATGTTTAAGAAGGTTGGTAAATTCCTTGCGGCTTTGGCAGCTATCCTGACGCTTGCGTATATTCTTGCGGTATACCCTCAAGTAGCACTAGTAGTAGTTGGCGCTTGTTACTTAGCGGCAGTGTGTGCTTGCGTGTGGAGTATAGTTAACTGGTAATACGACTCACTAAAGGAGGTACACACCATGATGTACTTAATGCCATTACTCATCGTCATTGTAGGATGCCTTGCGCTCCACTGTAGCGATGATGATATGCCAGATGGTCACGCTTAATACGACTCACTAAAGGAGacac            <  1:190699/301‑1 (MQ=255)`  
                                                                                                                                                                                                                                                                                                          `ttttAAGAAGGTTTGTAAATTCCTTGCGGCTTTGGCAGCTATCCTGACGCTTGCGTATATTCTTGCGGTATACCCTCAAGTAGCACTAGTAGTAGTTGGCGCTTGTTACTTAGCGGCAGTGTGTGCTTGCGTGTGGAGTATAGTTAACTGGTAATACGACTCACTAAAGGAGGTACACACCATGATGTACTTAATGCCATTACTCATCGTCATTGTAGGATGCCTTGCGCTCCACTGTAGCGATGATGATATGCCAGATGGTCACGCTTAATACGACTCACTAAAGGAGACACTATATGtt    <  1:611673/300‑1 (MQ=255)`  

                                                                                                                                                                                                                                                                                                             `|`                                                                                                                                                                                                                                                                                                             
`CCACGAATATCTCTCGCGCCTTAATGGATGAGTTCACTGAGACAGTAAAAGAGGCCACCCTAAGTCAATGGGGATTCTTTAGCCCATACGGTATTGGCGACAACGATGCTTGTACTATTAACCCTTACGATGGCTGGGCGTGTCAAATTAGCTACATGGAGGAAACACCTGATGGCTCTTTGCGGCACCCATCGTTCGTAATGTTCCGTGGCACCGA‑GGACAACCCTCAAGAGAAAATGTAATCACACTGGCTCACCTTCGGGTGGGCCTTTCTGCGTTTATAAGGAGACACTTTATGTTTAAGAAGGTTGGTAAATTCCTTGCGGCTTTGGCAGCTATCCTGACGCTTGCGTATATTCTTGCGGTATACCCTCAAGTAGCACTAGTAGTAGTTGGCGCTTGTTACTTAGCGGCAGTGTGTGCTTGCGTGTGGAGTATAGTTAACTGGTAATACGACTCACTAAAGGAGGTACACACCATGATGTACTTAATGCCATTACTCATCGTCATTGTAGGATGCCTTGCGCTCCACTGTAGCGATGATGATATGCCAGATGGTCACGCTTAATACGACTCACTAAAGGAGACACTATATGTTTC  >  NC_001604.1/7313‑7912`

  

Alignment Legend

Aligned base mismatch/match (shaded by quality score): `ATCG/ATCG` `< 3 ≤` `ATCG/ATCG` `< 14 ≤` `ATCG/ATCG` `< 27 ≤` `ATCG/ATCG` `< 36 ≤` `ATCG/ATCG` `< 38 ≤` `ATCG/ATCG`

Unaligned base: `atcg`    Masked matching base: `atcg`    Alignment gap: `‑`    Deleted base: `‑`

Reads not counted as support for junction

read\_name Not counted due to insufficient overlap past the breakpoint.

read\_name Not counted due to not crossing MOB target site duplication.
